# In Memoriam

**Published:** 1897-03

**Authors:** 


### In Memoriam.

### DR. JAMES A. SWASEY.

WE are called upon to mourn the death of one whose face will no longer be seen in our midst, Dr. James Atwood Swasey, for many years an active member of this society.

This sad event occurred early in the morning of December 24, 1896, at his residence, 1317 Michigan Avenue, Chicago.

Dr. Swasey had been in his usual good health up to about the middle of November, when he first noticed that he was suddenly breaking, and for a few days he went to the West Barden Springs; but not finding the desired relief, he returned to his summer home in Michigan, and from thence came to Chicago, where he died surrounded by his family and friends.

Dr. Swasey was the President of this society when the twenty-fifth anniversary was celebrated in 1889. He was President of the Odontological Society of Chicago in 1894–95, a member of the Illinois State Dental Society, the American Dental Association, and a member of the first International Dental Congress, Paris, France, 1889. He was also the first President of the Chicago College of Dental Surgery, and was re-elected for several years. He was an honorary member of several dental societies, State and local, in the United States.

The society loses one of its best representatives in the death of Dr. Swasey.

He was a man of strong character, high-minded and generous, with a pleasing manner, modest in his estimate of his own acquirements, ever ready to counsel and assist others.

He was a firm friend, a strong partisan, energetic and industrious, an inventor of many useful appliances, and devoted to his profession to the last.

We will miss his familiar face and hearty grasp of the hand in all of our subsequent sessions.

We mourn with his family in this hour of affliction, and extend our sympathies.

We place these lines of respect to his memory in our journal records, with the thought that his life had been a useful one to the community where he had resided for so many years, and with the ever-present hope and belief in the immortality of his spirit forever and forever.

*Be it resolved*, That a copy of this tribute be sent to his family, and others to the dental journals for publication.

A. W. Harlan,
Truman W. Brophy,
F. H. Gardiner,
*Committee.*

